# Enhancement of Electrical Safe Operation Area of 60 V nLDMOS by Engineering of Reduced Surface Electrical Field in the Drift Region

**DOI:** 10.3390/mi15070815

**Published:** 2024-06-24

**Authors:** Lianjie Li, Bao Zhu, Xiaohan Wu, Shijin Ding

**Affiliations:** School of Microelectronics, Fudan University, Shanghai 200433, China; 20112020013@fudan.edu.cn (L.L.); wuxiaohan@fudan.edu.cn (X.W.)

**Keywords:** electrical safe operation area, LDMOS, TCAD, BV_on_, Resurf

## Abstract

To enhance the electrical safe operation area (eSOA) of laterally diffused metal oxide semiconductor (LDMOS) transistors, a novel reduced surface electric field (Resurf) structure in the n-drift region is proposed, which was fabricated by ion implantation at the surface of the LDMOS drift region and by drift region dimension optimization. Technology computer-aided design (TCAD) simulations show that the optimal value of Resurf ion implantation dose 1 × 10^12^ cm^−2^ can reduce the surface electric field in the n-drift region effectively, thereby improving the ON-state breakdown voltage of the device (BV_on_). In addition, the extended n-drift region length of the L_d_ design also improves device BV_on_ significantly, and is aimed at reducing the current density and the electric field, and eventually suppressing the n-drift region impact ionization. The results show that the novel 60 V nLDMOS has a competitive BV_on_ performance of 106.9 V, which is about 20% higher than that of the conventional one. Meanwhile, the OFF-state breakdown voltage of the device (BV_off_) of 88.4 V and the specific ON-resistance (R_ON,sp_) of 129.7 mΩ⋅mm^2^ exhibit only a slight sacrifice.

## 1. Introduction

Recently, high-performance silicon-based LDMOS transistors featuring low specific R_ON,sp_, high BV_off_ [1,2], have been studied for various power management modes such as DC-DC converters [3], display drivers [4], and wireless chargers [5]. In particular, the LDMOS device with a high eSOA is an effective candidate for high operating voltage and high-reliability applications such as automotive electronic fields [6]. In practice, the BV_on_ is usually regarded as an evaluation criterion for the eSOA capability of a device. In general, the BV_on_ of LDMOS is determined by the Kirk effect, which gives rise to the depletion region broadening under the high-voltage and large-current conditions in the drift region, and parasitic bipolar junction transistor (p-BJT) turning on at the source and bulk regions. To enhance BV_on_, most LDMOS devices are fabricated by heavy doping in the source and bulk region to reduce the parasitic resistance (R_B_) and suppress the formation of p-BJT [7,8,9,10,11,12]. However, the resulting LDMOS devices have a higher threshold voltage, which deteriorates the device switching efficiency and enhances manufacturing difficulty due to the high-energy ion implantation. Therefore, some researchers have proposed optimizing the drift region structure or doping concentration to suppress the Kirk effect, thus manufacturing LDMOS with a high BV_on_ [13,14,15,16,17].

Taur et al. used higher doping concentrations in the n-drift region of LDMOS to push the triggering of the Kirk effect to higher current levels to improve the BV_on_ [12]. However, increasing the total doping concentration in the n-drift region also degrades BV_off_ performance [15]. Therefore, selectively reducing the surface electric field implantation was proposed to improve the BV_on_; however, the BV_off_ will also be degraded if the Resurf ion implantation is not well-controlled [16]. The TCAD simulation analysis shows that the BV_on_ is affected by not only the Kirk effect or the parasitic BJT characteristics, but also by the impact ionization weak point at the drift region of the device such as the field oxide corner or the surface near the drain side. As a result, it enables optimization of the impact ionization in the STI corner by adjusting the L_d_, and at the drain side by Resurf ion implantation doping and dimensions, thus achieving the optimized BV_on_ while reducing the impact on the device V_th_, BV_off_, and R_ON,sp_.

In this work, a novel LDMOS structure with reduced surface field implantation in the n-drift region and an optimized drift region length is proposed that can effectively improve the BV_on_ performance of the device. TCAD simulation results demonstrated that the current LDMOS device can exhibit a significantly superior tradeoff between BV_on_, BV_off_ and R_ON,sp_ than that of the conventional one.

## 2. Materials and Methods

**Figure 1:**
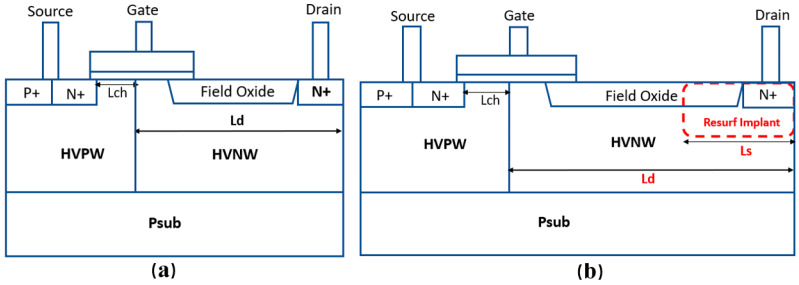
Schematic structures of: (**a**) the conventional 60 V nLDMOS; and (**b**) the proposed 60 V nLDMOS with Resurf engineering.

Figure 1a,b shows the cross-sectional schematic diagrams of the conventional LDMOS and our proposed one, respectively. For the conventional structure, the effective lengths of the channel region L_ch_ and the L_d_ are 1 and 9 μm, respectively. Meanwhile, the length of the field oxide is 1.2 μm, and the thicknesses of the gate oxide and shallow trench isolation (STI) are 12 nm and 0.4 μm, respectively. For the proposed structure, the L_ch_ and L_d_ are the same as the conventional structure; the differences lie in the Resurf ion implantation in the n-drift region and the critical dimensions of the L_d_ and L_s_ design. The threshold voltage (V_th_) of both LDMOS devices is 1 V.

Referring to Figure 1b, the main fabrication process of the proposed LDMOS is illustrated as follows. First, the p type single crystalline (100) silicon wafer with a resistivity of 10 Ω·cm is chosen as the substrate. Subsequently, the active area is defined by the field oxide, i.e., STI. Afterwards, the device channel, drift region and Resurf ion implantation region are formed by ion implantation with an implantation energy ranging from 100 to 1000 KeV and an implantation dose of 1 × 10^14^ cm^−2^. Then, the poly gate is defined by etching; the source and drain regions are generated by ion implantation with an implantation dose ranging from 1 × 10^14^ to 1 × 10^15^ cm^−2^, and an energy range of 10 to 100 KeV. Finally, the device contact, metal connection and silicon surface passivation are implemented.

## 3. Results

**Figure 2:**
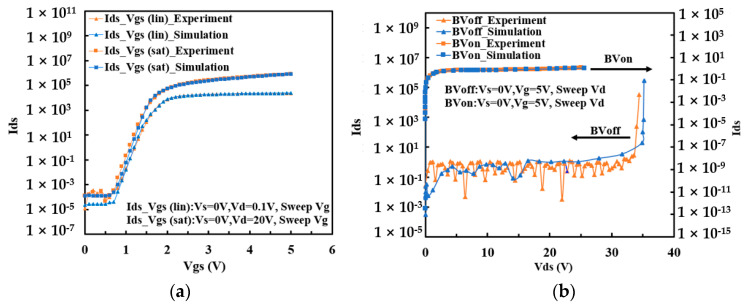
Calibration of simulated and experimental (**a**) transfer curve I_ds_-V_gs;_ and (**b**) BV_off_, BV_on_ curves of conventional LDMOS without Resurf implantation and dimension optimization.

In order to assure the accuracy of the subsequent TCAD simulation of 60 V nLDMOS, the simulation result was first calibrated in light of the experimental one for the 20 V LDMOS. Figure 2a shows that the transfer curves of 20 V nLDMOS, including I_ds_ -V_gs(lin)_ and I_ds_ -V_gs(sat)_, are well-calibrated by adjusting the mobility and impact ionization models during the TCAD simulation, while the breakdown characteristic curves I_ds_ -V_ds_ for OFF-state and ON-state are also calibrated, as shown in Figure 2b. It is worth noting that the I_ds_ -V_gs (lin)_ and I_ds_ -V_gs (sat)_ curves are normalized based on the I_ds_ of V_th_ = 1 V, and the BV_on_ and BV_off_ current are normalized based on the I_ds_ of V_ds_ = 20 V. As a result, the simulation results are highly consistent with the experimental results through the impact ionization and mobility model optimization. Finally, the TCAD simulation was implemented to the conventional structure of 60 V nLDMOS, and the main electrical parameters were obtained as BV_on_ of 88 V, BV_off_ of 93 V, and R_ON,sp_ of 116 mΩ·mm^2^. This provided an important reference for the novel design of the 60 V nLDMOS.

### 3.1. Effect of Resurf Ion Implantation Dose and Energy on BV_on_, BV_off_, and R_ON,sp_

**Figure 3:**
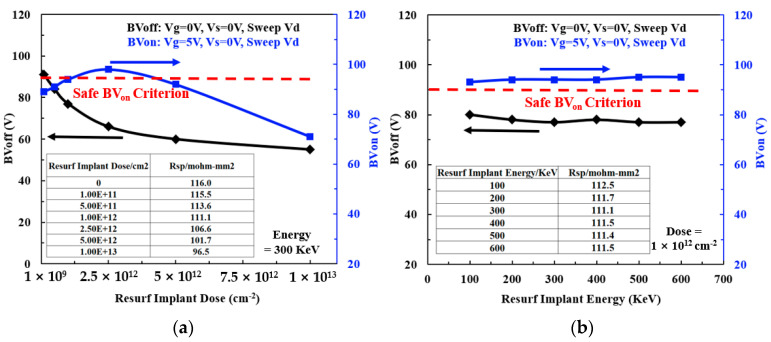
Dependence of BV_off_, BV_on_ and R_ON,sp_ on: (**a**) Resurf ion implantation dose; and (**b**) Resurf ion implantation energy, respectively, for the LDMOS devices with L_d_ of 9 μm, and L_s_ of 3 μm.

Figure 3a shows the effect of Resurf ion implantation dose on the BV_on_, BV_off_, and R_ON,sp_ while fixing the Resurf ion implantation energy at 300 KeV for the LDMOS devices with L_ch_ = 1 μm and L_d_ = 9 μm. When the Resurf ion implantation dose increases from 0 to 2.5 × 10^12^ cm^−2^, the resulting BV_on_ increases from 88 V to a maximum value of 98 V, and then decreases to 71 V when increasing the Resurf ion implantation dose to 1 × 10^13^ cm^−2^. This phenomenon can be explained by the fact that as the Resurf ion implantation dose increases, it can effectively suppress the strong electrical field caused by the Kirk effect, thereby increasing BV_on_ [15]. However, as the Resurf ion implantation dose further increases, the doping concentration in the Resurf region also increases. This will result in a higher current density, which enables the Kirk effect to be triggered, thus provoking the reduction in BV_on_. On the other hand, as the Resurf ion implantation dose increases, the resulting BV_off_ decreases gradually from 93 V to 55 V (i.e., a nearly 50% drop), which is completely different to the variation trend of BV_on_. This is mainly due to different electric fields across the drift region in the ON-state and OFF-state of the device. In other words, the resulting depletion region becomes narrower when the Resurf ion implantation dose increases, making it more likely that a stronger electric field in the drift region will occur, and a continuous decrease in BV_off_. In addition, the R_ON,sp_ also decreases gradually from 116 to 96.5 mΩ⋅mm^2^ with the increment of the Resurf ion implantation. This is mainly attributed to the enhanced doping in the n-drift region. Considering the essential requirements (BV_off_ > 80 V, BV_on_ > 90 V) of device safety operation, the ion implantation dose of 1 × 10^12^ cm^−2^ was chosen as the optimal condition, corresponding to BV_on_ = 94 V and BV_off_ = 77 V. Subsequently, the influence of the Resurf ion implantation energy on the BV_on_, BV_off_, and R_ON,sp_ was further studied in the case of a constant ion implantation dose of 1 × 10^12^ cm^−2^, as shown in Figure 3b. As the Resurf ion implantation energy increases from 100 to 600 KeV, the BV_on_ exhibits a small change between 93 to 95 V, and the BV_off_ also changes slightly from 80 to 77 V. In addition, the R_ON,sp_ remains unchanged at around 112 mΩ⋅mm^2^. The above results indicate that the Resurf ion implantation energy has little effect on the BV_on_, BV_off_, and R_ON,sp_ of the nLDMOS device. This can be ascribed to the thermal processes following the Resurf ion implantation, including the formation of field oxide and the thermal diffusion of the dopant at 1100 °C for 30 min, which could result in similar profiles of dopants under different implantation energies. In order to eliminate the influence of the thermal diffusion process on the Resurf ion implantation of the device, the Resurf implantation was carried out after the field oxidation formation and the thermal diffusion. From the TCAD simulation, it can be seen that the electrical results obtained are 89 V for BV_on_ and 93 V for BV_off_, which are close to the device performance of the 60 V nLDMOS conventional structure (88 V for BV_on_ and 93 V for BV_off_). This means that the Resurf implantation ions cannot effectively penetrate the field oxide into the silicon substrate surface, and no significant impact is present on the device characteristics. In addition, if the field oxidation is removed from the n-drift region of the device, a high electric field will appear on the surface of the device, thereby contributing to a significant decrease in the breakdown voltage of the device. As a result, the optimal Resurf ion implant energy of 300 KeV was chosen.

**Figure 4:**
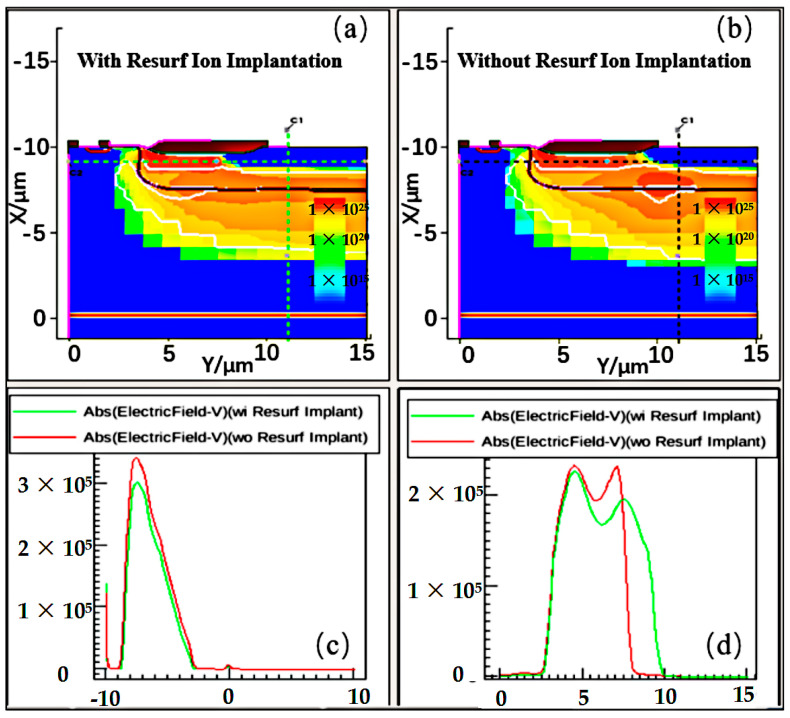
(**a**,**b**) Impact ionization rate distribution; and (**c**,**d**) the longitudinal and horizontal electric field of novel and conventional nLDMOS under the Resurf ion implantation condition at 300 KeV, 1 × 10^12^ cm^−2^.

Figure 4a,b shows the optimized Resurf ion implantation conditions on the surface of the n-drift region (300 KeV, 1 × 10^12^ cm^−2^) that could reduce the impaction ionization rate of the device surface effectively under the same bias condition. Furthermore, Figure 4c,d shows that the optimized Resurf ion implantation on the surface of the n-drift region also reduces the horizontal and vertical electric fields of the device surface significantly. In a word, the weakened impaction ionization rate and surface electric field assist in suppressing the Kirk effect near the drain terminal of the device, thereby improving the device’s ability to withstand large currents and high voltages, and ultimately achieving the optimization of device BV_on_.

### 3.2. Effect of Resurf Ion Implantation Length L_s_ and the n-Drift Region Length L_d_ on BV_on_, BV_off_, and R_ON,sp_

**Figure 5:**
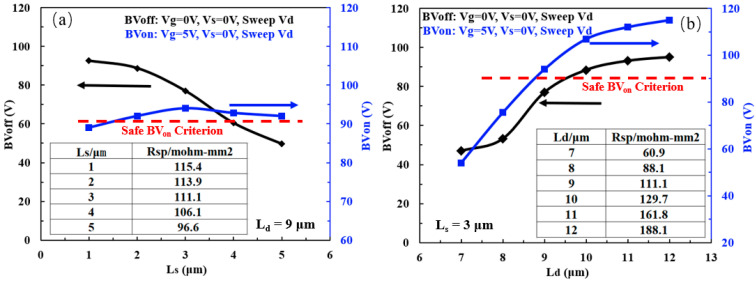
Dependence of BV_off_, BV_on_ and R_ON,sp_ on: (**a**) the Resurf ion implantation length Ls; and (**b**) L_d_ respectively, for the LDMOS devices with the Resurf ion implantation condition as 300 KeV, 1 × 10^12^ cm^−2^.

Figure 5a shows the dependence of BV_off_, BV_on_ and R_ON,sp_ on the Resurf ion implantation length L_s_ under the Resurf ion implantation conditions (300 KeV, 1 × 10^12^ cm^−2^), and the drift region length L_d_ of 9 μm. When L_s_ increases from 1 to 5 μm, the BV_off_ decreases from 92.5 to 49.6 V rapidly, with a significant change of nearly 50%. At the same time, when L_s_ increases from 1 to 3 μm, BV_on_ increases from 89 V to a maximum of 94 V, and then decreases to 92 V as L_s_ further increases to 5 μm. As a result, the change in BV_on_ is only 5 V which is similar to the effect of the Resurf ion implantation dose on device BV_on_. This phenomenon could be explained by the fact that it can effectively suppress the high electric field caused by the Kirk effect as L_s_ increases, thereby increasing BV_on_ [18]. However, a new high electric field will be generated as L_s_ further increases due to the higher doping concentration, which in turn will cause BV_on_ to start to decrease. Therefore, there is a maximum value for BV_on_ corresponding to the optimal L_s_ of 3 μm. In addition, R_ON,sp_ decreases from 115.4 to 96.6 mΩ⋅mm^2^ with the increase of L_s_, representing nearly 20% deceasing. As L_s_ increases, the region of high doping concentration in the n-drift region also increases, thus leading to the suppression of the electric field distribution in the OFF-state, and a significant decrease in BV_off_. In addition, due to the increase of doping concentration in the n-drift region, the resistance in the n-drift region correspondingly decreases, thereby resulting in a significant decrease in the R_ON,sp_ from 115.4 to 96.5 mΩ⋅mm^2^ with L_s_ increasing from 1 to 5 μm.

Figure 5b shows the effect of the n-drift region length L_d_ on the BV_off_, BV_on_ and R_ON,sp_ under the L_s_ of 3 μm. As L_d_ increases from 7 to 12 μm, the BV_on_ increases from 54 to 115 V, nearly a double enhancement. Meanwhile, the BV_off_ also has a significant improvement from 47 to 95 V. However, the R_ON,sp_ also increases rapidly. Therefore, L_d_ has a significant impact on device parameters such as BV_on_, BV_off_, and R_ON,sp_. Under a high gate voltage condition, such as V_gs_ = 5 V, the longer the n-drift region is, and the lower the conduction current of the device, making it more resistant to triggering of the Kirk effect, thereby improving the BV_on_. In addition, it is also conducive to a more uniform distribution of the electric field in the n-drift region when the device is turned off, as the length of the n-drift region increases, resulting in an increase in BV_off_. Finally, as the length of the n-drift region increases, it will increase the resistance of the n-drift region, which is responsible for a weakening of the device’s conduction current ability and an increase in the specific conduction resistance.

## 4. Discussion

**Figure 6:**
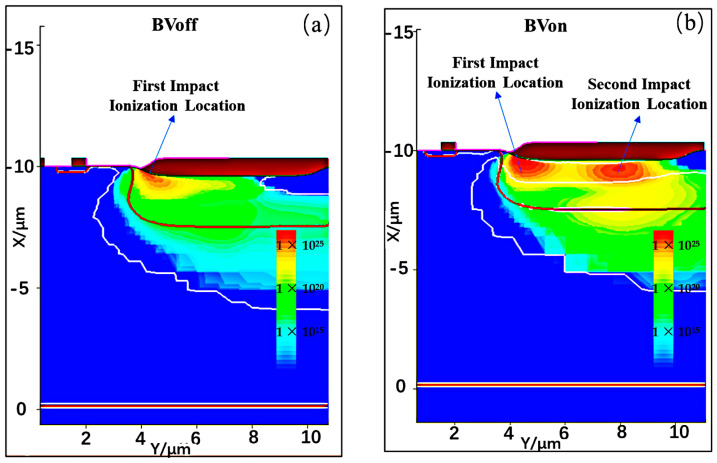
(**a**) Impact ionization position corresponding to device BV_off_; and (**b**) device BV_on_ under the L_d_ = 10 μm.

An interesting phenomenon is found in the TCAD optimization design of the n-drift region length L_d_. When L_d_ is increased to 10 μm, the BV_on_ and BV_off_ of the device begin to saturate. It is worth noting that the BV_off_ is determined by the avalanche impact ionization under the off state, while the BV_on_ is determined by the impact ionization under the on state of the large current. As a result, the underlying mechanisms affecting BV_on_ and BV_off_ are different. In Figure 6a, the factors affecting the BV_off_ saturation include the generation of the first avalanche impact ionization position and the depletion region broadening in the horizontal direction. When L_d_ increases to a certain extent, the width of the horizontal depletion region reaches its maximum, and the critical electric field of the avalanche occurs at the first avalanche impact ionization position. The BV_off_ is determined by the first avalanche impact ionization position. When L_d_ continues to increase, it no longer affects the distribution broadening of the depletion region and the avalanche impact ionization. As a result, the BV_off_ is no longer related to L_d_. In Figure 6b, the factors that contribute to the saturation of BV_on_ include the generation of the first avalanche impact ionization position and the generation of the second avalanche impact ionization position. When L_d_ increases to a certain extent, the electric field at the second avalanche impact ionization position has not yet reached the avalanche critical electric field; however, the first avalanche impact ionization position has already reached the avalanche critical electric field [19,20]. This means that BV_on_ is ultimately determined by the position of the first high electric field, and that the BV_on_ reaches its maximum value. Regarding the overall device performance, including optimal BV_on_, BV_off_, and R_ON,sp_, the L_d_ of 10 μm is chosen as the optimal condition. In this condition, the BV_on_ will increase to 106.9 V and BV_off_ will increase to 88.4 V, but R_ON,sp_ will sacrifice to 129.7 mΩ⋅mm^2^.

**Figure 7:**
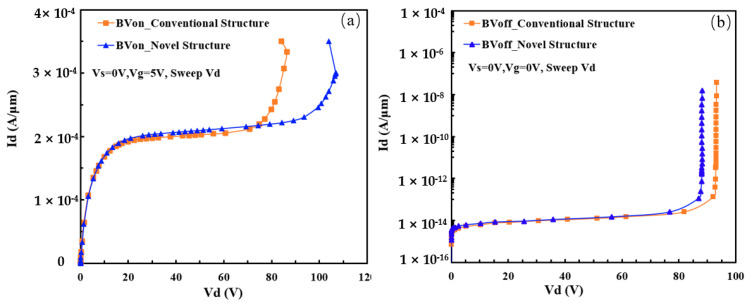
Comparison of (**a**) BV_on_ and (**b**) BV_off_ between 60 V nLDMOS novel structure and conventional one.

Finally, the electrical performance of the novel designed 60 V nLDMOS is 106.9 V for BV_on_, 88.4 V for BV_off_, and 129.7 mΩ⋅mm^2^ for R_ON,sp_, and the BV_on_ is nearly 20% higher than that of conventional device structures, and there is a slight sacrifice for BVoff and R_ON,sp_, as shown in Figure 7a,b. This result indicates that the Resurf ion implantation and the n-drift region dimension design is an effective method for improving the performance of device BV_on_.

## 5. Conclusions

In summary, a novel 60 V nLDMOS with Resurf engineering in the n-drift region was proposed that enhances the device electrical safe operation area by suppressing the Kirk effect. The TCAD simulations showed that an optimized Resurf ion implantation can effectively reduce the surface electric field in the n-drift region. Furthermore, for the design of key dimensions of the device, such as the n-drift region length L_d_, the device BV_on_ was significantly improved. Finally, the electrical performance of the novel designed 60 V nLDMOS optimized by Resurf ion implantation and device dimension design was optimized with 106.9 V for BV_on_, 88.4 V for BV_off_, and 129.7 mΩ⋅mm^2^ for R_ON,sp_. The novel structure BV_on_ is 20% higher than that of the conventional one, combined with a slight sacrifice on BV_off_ and R_ON,sp_. This work provides a very useful method for manufacturing high-reliability nLDMOS.

## Data Availability

The original contributions presented in the study are included in the article. Further inquiries can be directed to the corresponding authors.
